# Effect of nutrition education on hemoglobin level in pregnant women: A quasi-experimental study

**DOI:** 10.1371/journal.pone.0213982

**Published:** 2019-03-21

**Authors:** Dev Ram Sunuwar, Raj Kumar Sangroula, Nani Shova Shakya, Renu Yadav, Narendra Kumar Chaudhary, Pranil Man Singh Pradhan

**Affiliations:** 1 Armed Police Force Hospital, Kathmandu, Nepal; 2 Nepal Public Health Foundation, Kathmandu, Nepal; 3 Institute of Medicine, Tribhuvan University Teaching Hospital, Kathmandu, Nepal; 4 Nepal Orthopedic Hospital, Kathmandu, Nepal; 5 Department of Community Medicine and Public Health, Institute of Medicine, Tribhuvan University, Kathmandu, Nepal; CSIR-Foood Research Institute, GHANA

## Abstract

**Background:**

Anemia during pregnancy is a major public health problem globally with multiple causes including inadequate dietary intakes. The aim of the study was to assess the effect of nutrition education on nutritional knowledge, hemoglobin level and dietary intake of anemic pregnant women.

**Materials and methods:**

A quasi-experimental study was conducted among 115 mild to moderately anemic pregnant women attending ante natal clinics. Pregnant women were consecutively enrolled and assigned to receive nutrition education and diet plan in intervention group (n = 58) and general education only in control group (n = 57). The nutrition education was given to pregnant women on individual basis at the time of enrollment and follow-ups were done through biweekly phone calls and every 4 weeks during ANC visits. Baseline data were collected using semi-structure questionnaire for interview and hemoglobin level was also measured. Data were collected after 10 weeks of nutrition education intervention. Independent sample t-test was used to compare differences between the two groups.

**Results:**

Out of 115 pregnant women enrolled, 107 completed the study (Intervention: 53; Control: 54). At the end of the nutrition education intervention and iron rich food based diet plan, the change in hemoglobin level was significantly high in the intervention over control group [0.56±0.40gm/dl vs. 0.16±0.82gm/dl, p = 0.002]. The change in the maternal nutritional knowledge score on anemia and iron rich foods was significantly high in the intervention over control group [8.26±4.57 vs. 1.05±6.59, p<0.001].Consumption of iron rich food was significantly high in the intervention group (P<0.05).

**Conclusion:**

Provision of nutrition education and iron rich food based diet plan was significantly associated with improved hemoglobin levels, improved dietary intake and nutritional knowledge on anemia and iron rich foods.

## Background

Nutrition education is the foundation for any program intended for nutritional improvement [1]. The knowledge about proper nutrition and balanced diet during pregnancy is considered important for the well-being of both mother and fetus [2]. During pregnancy nutritional problems can impact on both mother and fetus, thus special attention is needed [3]. Inadequate diet during pregnancy can lead to various nutritional deficiencies such as anemia. Thus proper nutrition is a crucial part of pregnancy that should not be neglected [4].

Anemia during pregnancy is defined by the Centers of Disease Control and Prevention (CDC) and World Health Organization (WHO) as a hemoglobin concentration less than 11 g/dl. It is considered severe when hemoglobin concentration is less than 7.0 g/dl, moderate when hemoglobin falls between 7.0 and 9.9 g/dl, and mild when hemoglobin is from 10.0 to 10.9 g/dl [5].It is the most common nutritional deficiency disorder in the world. It is a major health problem that affects 25–50% of the population of the world and approximately 50% of pregnant women. WHO has estimated that prevalence of anemia in pregnant women is 14% in developed and 51% in developing countries [6, 7]. Prevalence of anemia among pregnant women has been stagnant in the past decade (48% in Nepal Demographic Health Survey 2011 vs 46% in 2016) despite iron folic acid supplementation program implemented throughout the country [7, 8].

Mostly used strategy to improve nutritional status of pregnant women is nutrition education that emphasizes on maternal diet quality by increasing the diet diversity [9].However less research has been carried out regarding this subject in Nepal. The aim of this study was to assess the effect of nutrition education on hemoglobin level in pregnant women.

## Materials and methods

### Study design

**Fig 1:**
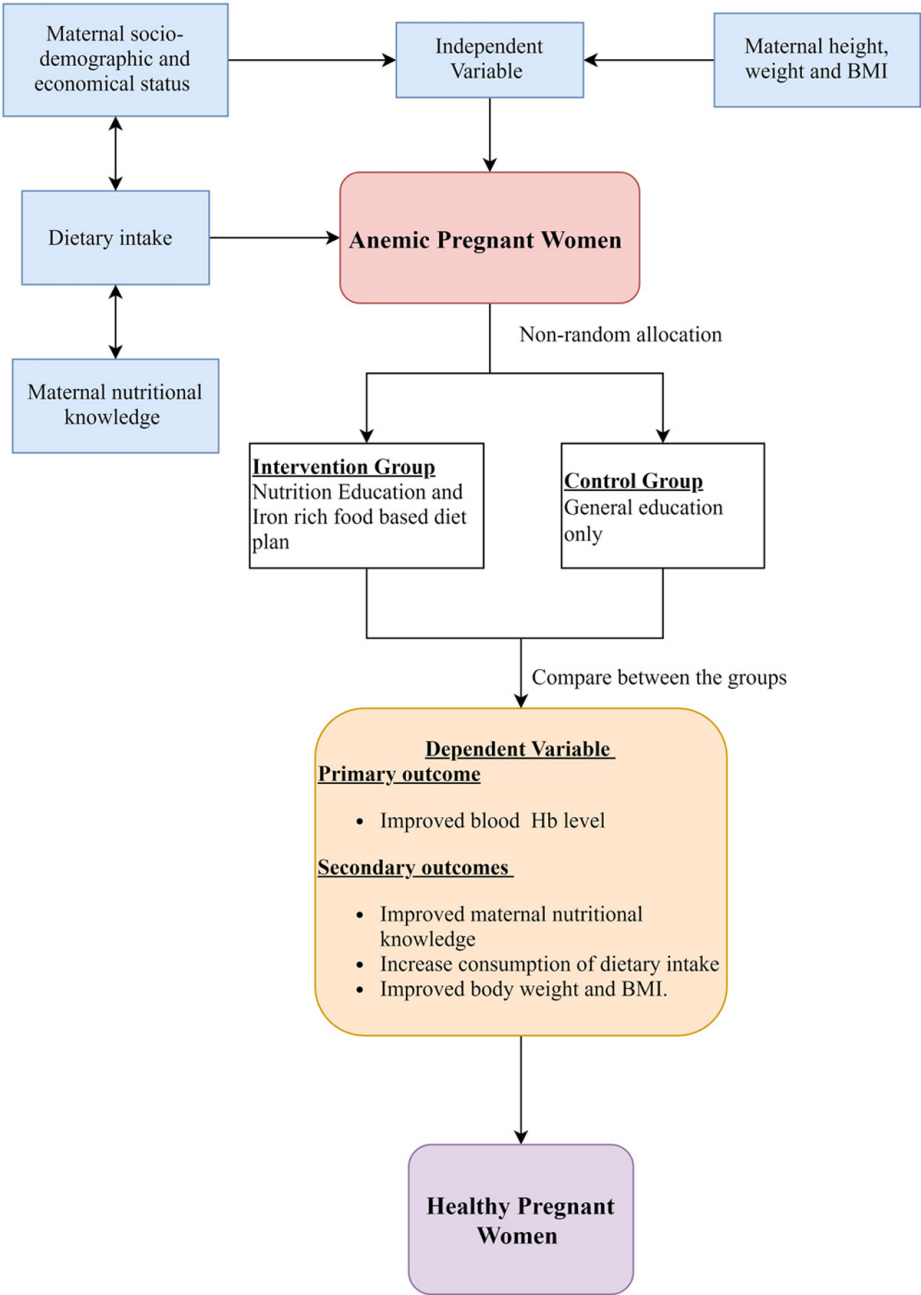
Conceptual framework of the study.

We conducted a quasi-experimental study among pregnant women who were in the second trimester (gestational age from 13 to 28 weeks) attending ante natal care (ANC) visits in Gynecology and Obstetrics outpatient department at Tribhuvan University Teaching Hospital (TUTH) in Kathmandu, Nepal. Pregnant women were consecutively enrolled and assigned to receive nutrition education and diet plan in the intervention group and general education only in the control group. End line data were collected after ten weeks of nutrition education intervention. The participants were enrolled from November 2017 to March 2018. End line and follow-up assessment were conducted between these periods. (Fig 1)

### Setting and sample

Tribhuvan University Teaching Hospital (TUTH) was selected purposively. Pregnant women aged 15–49 years who were willing to participate with mild and moderate anemia were included in the study. Pregnant women who were vegetarian and with special medical conditions like diabetes mellitus, hypertension, ante partum hemorrhage, renal disease, malignancy, cardio-pulmonary disease, hypo or hyperthyroidism, epilepsy and severe anemia were excluded from the study.

The sample size was calculated based on a similar study done by Otoo GE and Owusu WB in 2016 [10].After adjusting for 10% non-response rate and 20% loss through follow-up and drop out, the final sample size was 115 (58 participants in Intervention group and 57 participants in control group respectively) at 5% level of significance and 90% power. The sample size was calculated using test comparing two means in STATA version 13.0.

The trained data enumerator collected data from pregnant women who gave written informed consent to participate in the study. The baseline data collection technique was interview using semi-structured questionnaire. Anthropometric measurements like height, weight and body mass index (BMI), and biochemical assessment such as hemoglobin level estimation were done. End line data collection was done after ten weeks of nutrition education intervention. After baseline data collection was completed, participants were assigned to either the control or intervention group purposively. Pregnant women who attended in odd days were assigned to intervention group and those who attended the ANC visits in even days were assigned to control group.

Data enumerator was properly explained about the purpose of the study to the study participants in simple and understandable terms. Written informed consent was taken from participants before interview. Confidentiality and privacy of the participants was maintained. Ethical approval was obtained from the Institutional Review Board (IRB) of the Institute of Medicine (IOM) (Reference number 252(6-11-E) 2074/75).

### Nutrition education intervention

The intervention consisted of two nutrition education counseling sessions and iron rich food based diet plan to the intervention group and general education only to the control group during the study period. Nutrition education intervention was conducted at the Tribhuvan University Teaching Hospital (TUTH) for ten weeks. Nutrition education guideline on anemia with face-to-face lessons and written poster for individual counseling were developed and used for each pregnant woman. Key message on nutrition education to the intervention group consisted of causes of anemia, consequences of anemia in pregnancy, iron rich foods, enhancers and inhibitors of iron absorption and iron rich food based diet plan. General education to the control group consisted of hygiene and sanitation, rest and exercise, danger signs in pregnancy.

The nutrition education counseling sessions were held at the time of enrollment lasting approximately one hour. At the end of the session, information and education materials on anemia were distributed. Follow up was done every 4 weeks during ANC visits at the hospital. In every two weeks, phone call of about 3–5 minutes was done as follow-up to know the situation of pregnant women and convey key messages.

Diet plan was developed and used for pregnant women on an individual basis. Diet was planned by using food exchange list and portion size adopted by the dietary department of the Tribhuvan University Teaching Hospital (TUTH).Total iron value found in each food items per household measurement were calculated and incorporated in diet plan. Harris-Benedict’s equation was used to calculate total energy expenditure based on the ideal body weight. The total energy requirement was determined by adding extra 300–350 kcal required for pregnant women in second trimester. Dietary guideline was adopted from dietary guidelines by Indian Council for Medical Research 2010 [11]. Accordingly a balanced diet menu for each pregnant woman was planned. The total day’s menu plan was distributed into 5 meals as breakfast, lunch, snack, and dinner and bedtime snack with emphasis on iron rich foods. Diet plan was made at the time of enrollment for individual pregnant women in the intervention group and follow-up were done every 4 weeks during ANC visits and phone calls.

Maternal nutritional knowledge was assessed before and after intervention by interview using a semi-structure questionnaire. Participants were scored on the proportion of correct responses to questions on anemia and iron-rich foods. The minimum and maximum scores were 0 and 24 respectively. Scores from 0 to 7, 8 to 16 and 17 to 24 were considered as poor, average and good knowledge respectively [12].

### Outcome measure

The primary outcome for this study was the effect of nutrition education and diet plan on hemoglobin level of pregnant women after ten weeks of intervention. Secondary outcomes included the change in body weight, nutritional knowledge of mothers and dietary intake.

### Statistical analysis

Data entry was recorded using EpiData version 3.2 and analyzed by using STATA 13.0. Frequencies, percentage, mean (standard deviation) were calculated under descriptive statistics. Chi-square test was used to analyze association of categorical variables between intervention and control groups. Independent sample t-test assuming equal variance was used to compare between the intervention and control groups. All probability values less than 0.05 were considered to be statistically significant.

## Results

**Fig 2:**
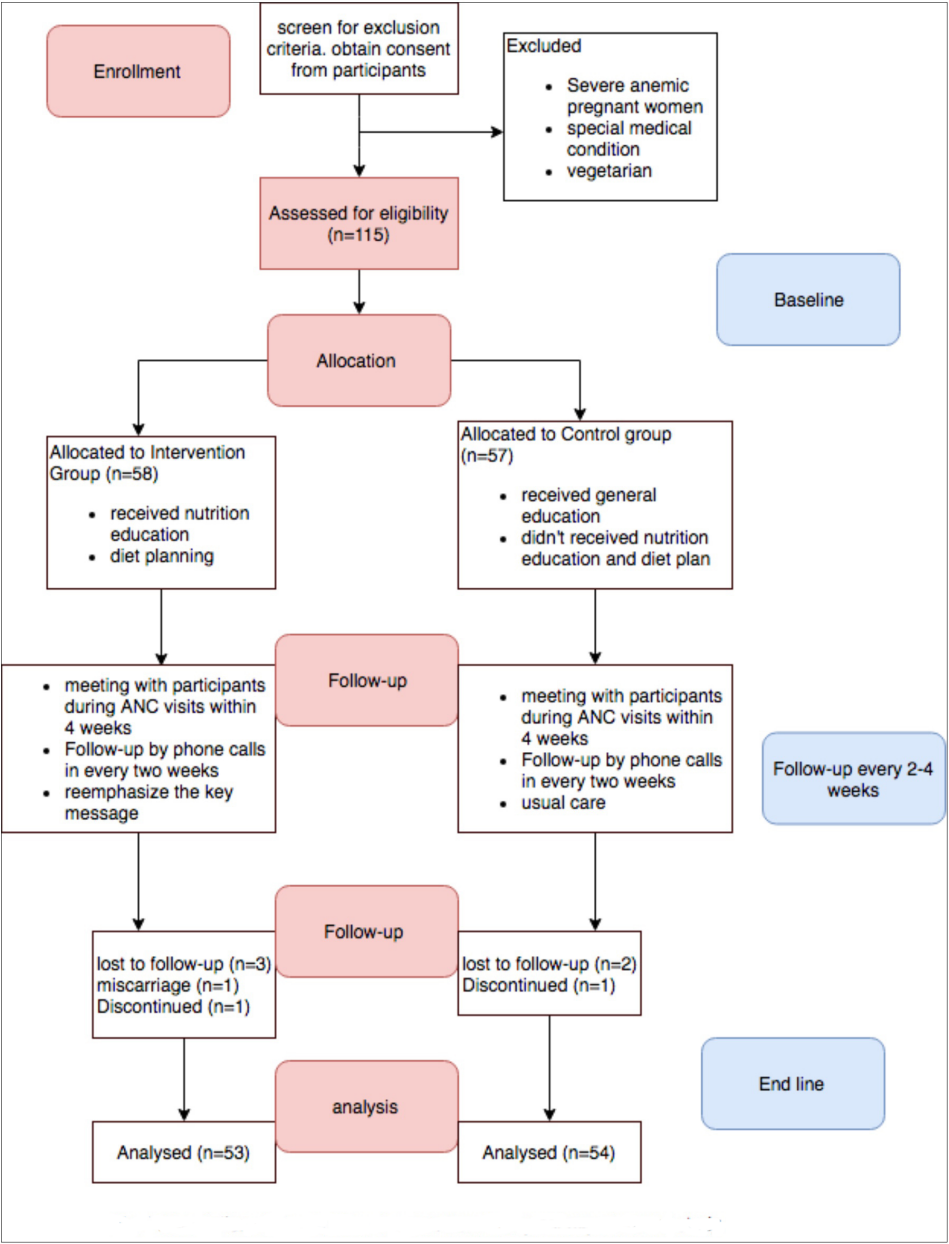
Flow diagram of the study.

In the study 58 pregnant women received nutrition education as well as iron rich food based diet plan, while 57 pregnant women received general education only. Eight pregnant women left the trial during the study. Among them five participants could not be followed up, two discontinued the study and one had abortion (Fig 2).

There was no significance difference between the intervention and control groups regarding age, parity, educational level, ethnicity, religion, occupation, ecological zone, and income level (Table 1).

**Table 1:** Socio-demographic information of pregnant women.

**Variables**		**Control Group** **(n = 54)**	**Intervention Group** **(n = 53)**	**p value**
		**Mean±SD**	**Mean±SD**	
**Mean age (years)**		26.06±4.98	26.23±4.74	0.856
**Base line gestational age(weeks)**		21.44±4.65	21.08±4.87	0.692
**End line gestational age (weeks)**		29.93±4.58	28.89±4.47	0.234
**Gravida**				
Primigravida		1.57±0.79	1.74±0.93	0.16
Multigravida		1.15±0.61	1.07±0.26	0.158
**Variables**		**Control Group(n = 54)**	**InterventionGroup(n = 53)**	
		**n(%)**	**n(%)**	
**Age groups**	15–19	3(5.6)	2(3.8)	0.595
	20–29	41(75.9)	39(73.6)	
	30–40	10(18.5)	12(22.6)	
**Education**	No education	4(7.4)	1(1.9)	0.662
	Literate only	1(1.9)	6(11.3)	
	Primary (1–5)	2(3.7)	2(3.8)	
	Secondary (6–9)	13(24.1)	10(18.9)	
	SLC & above	34(63.0)	34(64.2)	
**Religion**	Hindu	42(77.8)	42(79.2)	0.506
	Buddhist	6(11.1)	8(15.1)	
	Others	6(11.2)	3(5.7)	
**Occupation**	House wife	27(50.0)	32(60.4)	0.621
	Employee	12(22.2)	12(22.6)	
	Trader	8(14.8)	6(11.3)	
	Unemployed	2(3.7)	-	
	Others	5(9.3	3(5.7)	

This study found that a majority of pregnant women in both intervention and control groups had mild anemia (9–10.9 gm/dl) at baseline (85.2% and 96.3% respectively). After intervention, one third (34%) of pregnant women in intervention group and 9.3% in control group had normal level of hemoglobin (>11gm/dl). At the end of 10 weeks, the change in hemoglobin level was significantly higher in the intervention group compared to control group (0.56**±**0.40gm/dl vs 0.16 **±**0.82gm/dl, p = 0.002) (Table 2).

**Table 2:** Comparison of primary outcome variables for base line and end line in control and intervention groups.

Variables	Control group(n = 54)	Intervention group(n = 53)	p value^1^
	Mean ± SD	Mean ± SD	
**Blood Hb level gm/dl**			
Baseline	10.18**±**0.62	9.99**±**0.87	0.209
End line	10.35**±**0.64	10.55**±**0.79	0.144
Change	0.16**±**0.82	0.56**±**0.40	0.002*

**^1^** Independent sample t-test

*statistically significant is at p<0.05

Before intervention, only 14.8% in control group and 7.4% in intervention group had good nutritional knowledge score. After intervention, two third of the pregnant women (66.0%) in intervention group had good nutritional knowledge score on anemia and iron rich foods, compared to only one fourth (24.1%) in control group. There was significance difference in the nutritional knowledge score of pregnant women in the intervention group over control group at baseline (p<0.001) (Table 3)

**Table 3:** Comparison of secondary outcomes variables for base line and end line in control and intervention groups.

Variables	Control group(n = 54)	Intervention group(n = 53)	
	Mean ± SD	Mean ± SD	p value^1^
**Maternal nutritional knowledge score**			
Baseline	10.31**±**5.17	9.24±4.67	0.200
End line	11.37**±**5.02	17.37±3.25	<0.001*
Change	1.05±6.59	8.26±4.57	<0.001*

^**1**^ Independent sample t-test

*statistically significant is at p<0.05

There was no significant difference in weight and BMI between the groups post intervention (Table 4).

**Table 4:** Comparison of secondary outcomes variables for base line and end line in control and intervention groups.

Variables	Control group(n = 54)	Intervention group(n = 53)	p value^1^
	Mean ± SD	Mean ± SD	
**Weight, kg**			
Baseline	54.87 ±8.81	52.68 ±7.43	0.167
End line	56.59 ±8.25	56.01 ±7.14	0.702
Change	1.79 ±9.84	3.28 ±8.31	0.401
**Height, cm**	154.11±5.11	153.47±5.77	0.541
**BMI, kg/m2**			
Baseline	22.87 ±3.86	22.18 ±3.15	0.322
End line	23.83 ±3.27	23.77 ±2.62	0.921
Change	0.95 ±4.92	1.58 ±2.80	0.419

^**1**^ Independent sample t-test

BMI = Body mass index (kg/m^2^)

*statistically significant is at p<0.05.

There was significantly higher intake of red meat, fish and liver (p<0.001), vitamin C rich fruits (p = 0.006), dairy products (p = 0.013), eggs (p = 0.016) and dark green vegetables (p<0.006) in the intervention compared to control group (Table 5).

**Table 5:** Comparison of dietary intake of selected food items between control and intervention groups using seven days food frequency questionnaire.

Variables	Control group(n = 54)	Intervention group(n = 53)	
Food groups	Mean ± SD	Mean ± SD	p value^1^
**Red meat, liver, fish**			
Base line	1.90 **±**1.17	1.93 **±**1.43	0.927
End line	1.93 **±**1.14	3.88 **±**1.82	0.001*
Change	0.03 **±**1.33	2.23 **±**1.85	<0.001*
**Vitamin C rich fruits**			
Base line	4.35 **±**2.11	4.20**±**2.14	0.729
End line	4.20 **±**2.26	5.42**±**2.01	0.005*
Change	0.04 **±**2.85	1.41 **±**2.07	0.006*
**Legumes and nuts**			
Base line	4.17**±**2.25	4.04 **±**2.55	0.800
End line	4.32 **±**2.17	4.72 **±**2.22	0.369
Change	0.21 **±**3.11	0.78 **±**2.17	0.314
**Dairy products**			
Base line	4.67 **±**2.62	4.55 **±**2.60	0.827
End line	4.92 **±**2.37	6.55 **±**1.51	<0.001*
Change	0.26 **±**3.13	2.04 **±**3.28	0.013*
**Cereal/Grains**			
Base line	6.98 **±**0.13	6.81**±**0.98	0.219
End line	6.87 **±**0.83	7.04 **±**0.89	0.315
Change	0.11 **±**0.86	0.19 **±**1.31	0.161
**Eggs**			
Base line	4.71 **±**2.56	3.28**±**2.42	0.016*
End line	4.02 **±**2.45	4.84 **±**2.37	0.097
Change	0.37 **±**3.80	1.89 **±**3.63	0.016*
**Vitamin A rich fruits**			
Base line	3.10 **±**2.18	2.60**±**1.94	0.302
End line	3.21 **±**1.92	3.57**±**2.03	0.384
Change	0.09 **±**2.69	1.05 **±**2.39	0.070
**Dark green leafy vegetables**			
Base line	6.08 **±**1.67	5.37**±**2.17	0.063
End line	5.80 **±**1.66	6.55 **±**1.17	0.008*
Change	0.20 **±**2.29	1.11 **±**2.57	0.006*

^**1**^ Independent sample t-test

* Statistical significance is at p<0.05

## Discussion

Present study indicates that the nutrition education and iron rich food based diet plan during pregnancy led to improved hemoglobin level, maternal weight gain and increased consumption of iron rich foods. At end line, there was significant improvement in the hemoglobin concentration of intervention group compared to control group in pregnant women. Similar result were reported by a randomized study done in the University of Ghana showing that nutrition education emphasizing iron rich foods consumption was positively associated with improved hemoglobin levels (CG, 0.1+1.3 vs. -0.7+1.4) [10]. A quasi experimental study done by Al-tell MA et al, (2010) indicated a significant positive relationship between dietary practices and improvement of hemoglobin level of pregnant women [13]. Similarly, in a pre-test post-test study in pregnancy done by Garg & Kashyap (2006), individual counseling significantly improved mean hemoglobin levels in pregnant women (0.97 vs. 1.58, P<0.001) [14]. Likewise, a randomized control trial among the Nepalese pregnant women, the education program only seen to be significantly higher hemoglobin change (0.23 gm/dl) compared to the control group (P<0.01) [15]. While a randomized control trial done in Greece showed no significant effect of nutrition education and counseling on hemoglobin level in the intervention group compared to control group [16].

A review of prior randomized control trial and a quasi-experimental studies reported substantial and significant effects when nutrition education and counseling was provided with nutritional supplements, mostly by way of micronutrients, compared to nutrition education alone [17]. Nepal Demographic and Health Survey reports showed that just 42% of women took the recommended dose of iron during pregnancy and 41% of women aged between 15 to 49 years were anemic [8]. Thus, compliance of iron supplementation is still low in pregnant women in Nepal. According to Multi-Sectoral Nutrition Plan II, Nepal has a target of reducing the prevalence of anemia among women of reproductive age by 50% in line with the Sustainable Development Goals [18]. Counseling on nutrition education and iron rich food based diet plan for the pregnant mothers could be an effective strategy to reduce anemia among the pregnant women.

The nutrition education intervention and iron rich food based diet plan was significantly associated with increase in maternal nutritional knowledge score of anemia and iron rich foods intake in intervention group compared to control group (66% Vs 24.1%). Randomized studies done in university of Ghana reported that significant increase in knowledge exhibited by the intervention group at the end of the intervention period [10]. The study done in Ethiopia revealed that the knowledge of pregnant women on nutrition during pregnancy significantly increased after the provision of nutrition education and the specific dietary practices [19].The intervention study design done in Kalyobia Governorate (Moshtoher, KafrShoukr, and Kaha) (n = 200) result showed 78% of pregnant women had attained good nutritional knowledge score after intervention [20]. However, mostly counseling done during ante natal visits tends to be general in the context of Nepal. Our finding suggests pregnant women who have good nutritional knowledge can have improved hemoglobin level. Thus, nutrition education and counseling during ante natal visits could improve maternal nutritional knowledge regarding iron rich foods. Similar result were reported by other study done, an interventional study that after nutrition education session, there was significant improvement in the nutritional knowledge score which can helped to prevent anemia [21].

The present study shows average maternal weight gain was higher in the intervention group compared to control group (3.28 kg vs. 1.79kg). Similar result was reported by quasi-experimental study done in the Indonesia (2014), that increase in average maternal weight gain in the treatment group higher than the control group (3.017kg vs. 1.80kg) [3].Another study conducted by Kafatos AG et al, indicated that nutrition counseling during pregnancy can improve dietary intake and maternal weight gain [16].

The present study result shows that pregnant women who received nutrition education and iron rich food based menu plan had a significant increase in the consumption level of red meat, fish liver, vitamin C rich fruits, dairy products, eggs and dark green vegetables compared to control group. The study done by Liu N et al, (2009) result also showed that increased fruits consumption in the intervention group than control group [22]. Pregnant women in the intervention group reflected behavioral change by practicing minimum of 3 or more meal consumption [23]. Nutrition education and counseling has been found in other studies to improve maternal diet including dietary practices and consumption of macro and micronutrients [10, 24]. Micronutrient deficiencies can lead to poor maternal health outcome and pregnancy associated complication [25]. Brough L et al, (2010) showed that increase consumption of micronutrient in pregnancy can improve nutritional status of pregnant women [26].A study carried out by Hasswane N et al, (2015) also revealed the need to implement educational program to improve nutritional knowledge and sensitization of women in order to ensure adequate intake of iron and increase iron bioavailability food [27].

Our study had several limitations. The sample size of our study was less therefore the findingscannot be generalized to a wider population. The compliance to nutrition education and iron richfood based diet plan was not assessed in this study. Only hemoglobin level was measuredtherefore we could not assess the differentiation of anemia, such as iron deficiency, nutritional,genetic and infectious anemia in this study. Participants were assigned to either the control orintervention group purposively. So, there was chance of bias due to lack of randomization.

## Conclusion

Our study concludes the provision of nutrition education and iron rich food based diet plan for pregnant women was found to be associated with improved hemoglobin level. Provision of nutrition education and iron rich food based diet plan was significantly associated with improved nutritional knowledge score and improved dietary intake.

## Supporting information

S1 File Questionnaire. (DOCX)

S2 File Dataset. (ZIP)
