# Supplementary material for: Effect of nutrition education on hemoglobin level in pregnant women: A quasi-experimental study
Source: PLoS One. 2019 Mar 21;14(3):e0213982. doi: 10.1371/journal.pone.0213982 (PMC6428266; doi:10.1371/journal.pone.0213982)
Supplement: S1 File — (DOCX) [file pone.0213982.s001.docx]

## Appendix 3: Main Study Questionnaire in English - Baseline/End line

Title of Thesis**: Effect of Nutrition Education on Haemoglobin Level in Pregnant Women: A Quasi experimental Study in one tertiary care Hospital in Kathmandu, Nepal.**

**Participant ID Number……………………. Date (dd/mm/yy) ……………….**

**Mobile number…………………………………………………..**

| **Q.N.** | **Questions** | **Response** | **Codes** | **Skip/Remarks** |
| --- | --- | --- | --- | --- |
| **Section 1: Socio-Demographic characteristics.** | | | | |
| **1** | Age | ……………years |  |  |
| 2 | How many times have you become pregnant? | …………… times |  |  |
| 3 | How many live deliveries have you had? |  |  |  |
| 4 | Gestational age ______ weeks (***copy from ANC attendance book)*** |  |  |  |
| 5 | What is your education level? | No education  Literate only  Primary(1-5)  Some secondary(6-9)  SLC and above | 1  2  3  4  5 |  |
| 6 | What is your caste/ethnicity? | Hill Brahmin  Hill Chhetri  Terai Brahmin/Cheetri  Other Terai Caste  Hill Dalit  Terai Dalit  Newar  Hill Janjati  Other, Specify……………….. | 1  2  3  4  5  6  7  8  99 |  |
| 7 | What is your religion? | Hindu  Buddhist  Muslim  Kirat  Christian  Others, Specify………… | 1  2  3  4  5  99 |  |
| 8 | What is your occupation? | House wife  Farmer  Employee  Trader  Unemployed | 1  2  3  4  5 |  |
| 9 | How much is your average monthly Income? | ………….. |  |  |
| 10 | Ecological region | Mountain  Hill  Terai | 1  2  3 |  |
| 11 | Residence | Rural  Urban | 1  2 | VDCs are rural, municipalities are urban. |

| **Q.N.** | | **Questions** | **Response** | **Codes** | | **Skip/Remarks** |
| --- | --- | --- | --- | --- | --- | --- |
| **Section 3:- Maternal nutrition knowledge – Baseline [ ]End line [ ]** | | | | | | |
| **1** | Have you heard of anemia? | | No  Yes | | 0  1 |  |
| **2** | What are the symptoms of anemia? (***Do not prompt. Allow her to mention and circle the options mentioned***) | | I don’t know  Pallor  Tiredness  Body weakness  Dizziness  Headache  Breathlessness  Rapid heartbeat  Other (specify)…... | | 0  1  2  3  4  5  6  7  99 |  |
| **3** | What causes anemia? | | I don’t know  Low iron (blood increasing) diets  Parasites  Heavy menstrual period  Others (specify)…………………………………….. | | 0  1  2  3  99 |  |
| **4** | Could you mention the foods that increases blood (iron-rich foods) | | No  yes | | 0  1 |  |
| **5** | If yes mention | | …………….. | |  |  |

**I am going to mention to you a list of foods. Please tell from the list those which enhance iron absorption and those which inhibit iron absorption?**

| Food item | **1= iron absorption enhance, 2 = iron absorption inhibits,**  **0 = I don’t know** |
| --- | --- |
| Tea |  |
| Red meat,liver |  |
| Orange,Amala,lemon |  |
| Coffee |  |
| Eggs |  |
| Fish |  |
| Organ meat |  |
| Grain cereals |  |
| Milk |  |
| Green leafy vegetables |  |
|  |  |

| **Q.N.** | **Questions** | **Response** | **Codes** | **Skip/Remarks** |
| --- | --- | --- | --- | --- |
| **7** | When sharing food at home, who do you think should be served more meat and other animal source foods? | I don’t know  Father  Mother  Pregnant mother  Child  Other (specify)………..  …………………. | 0  1  2  3  4  99 |  |
| **8** | Do you have any food beliefs/taboos related to pregnancy? | No  yes | 0  1 |  |
| **9** | If yes mention them |  |  |  |

**Section 4: FOOD FREQUENCY QUESTIONNAIRE– Baseline [ ] End line [ ]**

**(Ask): Did** you eat each item at least once in the least once in the last 24 hours or 7 days?

| **FOOD GROUP** | **Have you eaten this in the last 24 hours**  **1 = Yes 0 = No** | **If yes, how many times in the last 24 hours did you eat it? 1, 2 or 3, etc** | **Have you eaten this in the last seven days?**  **1 = Yes 0 = No** | **If yes, how many times in the last 7 days did you eat it? 1, 2,3, 4,5,6 or 7 etc** |
| --- | --- | --- | --- | --- |
| Red meat, liver |  |  |  |  |
| Fish |  |  |  |  |
| Vitamin C rich foods-citrus fruits |  |  |  |  |
| Legumes and nuts(beans, groundnuts, peanuts, soybeans etc) |  |  |  |  |
| Dairy products(milk, butter, yoghurt and cheese) |  |  |  |  |
| Grains, roots and tubers (rice, bread, maize, buckwheat, yam etc) |  |  |  |  |
| Eggs |  |  |  |  |
| Vitamin A rich fruits (mangoes, carrot, sweet potatoes, etc) |  |  |  |  |
| Dark green leafy vegetables |  |  |  |  |

**SECTION 5: ANTHROPOMETRY Baseline [ ] End line [ ]**

**SECTION 6: BIOCHEMICAL MEASUREMENT –Baseline [ ] End line [ ]**

## Appendix 4: Questionnaire in Nepali

**सहभागीको आई.डी.नं........................ मितिः-**

**नाम/थर........................................... मोबाईल नं.**

| **खण्ड १:सामाजिक तथा जनसाङ्ख्यिक विवरण** | | | | |
| --- | --- | --- | --- | --- |
| **सि.नं.** | **प्रश्नहरु** | **प्रतिक्रिया** | **कोड** | **स्कीप/टिप्पणी** |
|  | उमेर | ..............बर्ष |  |  |
|  | तपाई कती पटक गर्भवती हुनु भयो ? | ..............पटक |  | यदी एक पटक हो भने ४ मा जानुहोस |
|  | तपाईले कति जना जिवित बच्चा जन्माउनु भयो ? |  |  |  |
|  | गर्भवस्था उमेर (ANC कार्डबाट सार्नुहोस) ? | ..............हप्ता |  |  |
|  | तपाईले कति पढ्नु भएको छ ? | निरक्षर  सामान्य लेखपढ  प्राथमिक (१-५)  माध्यमिक (६-१०)  उच्च माध्यमिक र त्यो भन्दा बढी | १  २  ३  ४  ५ |  |
|  | जातजाती | पहाडी ब्राहमण  पहाडी क्षेत्री  तराई ब्राहमण /क्षेत्री  अन्य तराई जाती  पहाडी दलित  तराई दलित  नेवार  पहाडी जनजाती  अन्य (खुलाउनुहोस)............ | १  २  ३  ४  ५  ६  ७  ८  ९९ |  |
|  | तपाईले कुन धर्म मान्नु हुन्छ ? | हिन्दु  बौद्ध  मुस्लिम  किराँत  क्रिश्चियन  अन्य............ | १  २  ३  ४  ५  ९९ |  |
|  | तपाईको पेशा के हो ? | गृहणी  कृषि  नोकरी  ब्यापार  बेरोजगार  अन्य (खुलाउनुहोस)...... | १  २  ३  ४  ५  ९९ |  |
|  | तपाईको मासिक आम्दानी कति छ ? | रु १०¸००० भन्दा कम  रु १०¸००० देखि २५¸०००  रु २५¸००० देखि ५०¸०००  रु ५०¸००० भन्दा माथि | १  २  ३  ४ |  |
|  | तपाई कुन ठाउँमा बसोबास गर्दै आउनु भएको छ ? | हिमाल  पहाड  तराई | १  २  ३ |  |
|  | तपाई बसोबास गर्दै आउनु भएको ठाउँ कस्तो छ ? | ग्रामिण  शहर | १  २ | गाउँपालिका भनेको ग्रमिण र नगरपालीका भनेको शहर |

| **खण्ड ३:मातृपोषण ज्ञान** | | | | |
| --- | --- | --- | --- | --- |
| **सि.नं.** | **प्रश्नहरु** | **प्रतिक्रिया** | **कोड** | **स्कीप/टिप्पणी** |
|  | के तपाई रक्तअल्पताको बारेमा सुन्नु भएको छ ? | सुनेको छैन  सुनेको छु | ०  १ | यदि सुनेको छैन भने ४ मा जानुहोस |
|  | रक्तअल्पताका लक्ष्णहरु के के हुन ? (हतार नगर्नुहोस । उल्लेखित बिकल्पहरुमा उल्लेख गरि गोलो लगाउनुहोस ) | मलाई थाहा छैन  फिक्का हुनु  थकित हुनु  शरिर कमजोर हुनु  चक्कर लाग्नु  टाउको दुख्नु  स्वासप्रस्वास बढ्नु  मुटुको धडकन बढ्नु  अन्य ........... | ०  १  २  ३  ४  ५  ६  ७  ९९ |  |
|  | के कारणले रक्तअल्पता हुन्छ ? | मलाई थाहा छैन  आईरन युक्त खानाको कमि भएर  शरीरमा परजिबि भयो भने  महिनावारी हुदाँ धेरे रक्तस्राव भएमा  अन्य...... | ०  १  २  ३  ९९ |  |
|  | के तपाई त्यस्ता खानाहरुको नाम भन्न सक्नुहुन्छ जसले शरिरमा रगतको मात्रा बढाउँछ ? (आईरनयुक्त खाना) | सक्दिन  सक्छु | ०  १ |  |
|  | यदि सक्नु हुन्छ भने तिनिहरुको नाम भन्नुहोस ? |  |  |  |

|  | म तपाईलाई खानाहरुको सुचि/तालिका बताउन गईरहेको छु¸ ती मध्ये कुनचाहि खानाले शरीरमा आईरन शोषन बृद्धि गर्छ र कुन खानाले आईरन शोषन अबरोध गर्दछ ? | | | |
| --- | --- | --- | --- | --- |
| खानाको प्रकार | | ०= मलाई थाहा छैन  १=आईरन शोषन बृद्धि गर्छ  २=आईरन शोषन अबरोध गर्छ | | |
| चिया/कफि | |  | | |
| रातो मासु¸ कलेजो¸ माछा¸अण्डा | |  | | |
| सुन्तला¸ अमला¸ कागती | |  | | |
| गेडागुडी/अन्न | |  | | |
| दुध | |  | | |
| हरियो सागपात | |  | | |
| **सि.नं.** | **प्रश्नहरु** | **प्रतिक्रिया** | **कोड** | **स्कीप/टिप्पणी** |
|  | तपाईको बिचारमा घरमा खाना बाँड्दा कसलाई मासु र अन्य जनवारहरुको स्रोत बाट प्राप्त हुने खाना बढी दिनु पर्छ ? | मलाई थाहाँ छैन  बुबा  आमा  गर्भवती आमा  बच्चा  अन्य... | ०  १  २  ३  ४  ९९ |  |
|  | तपाइँसँग गर्भावस्थासँग सम्बन्धित कुनै पनि खाद्य प्रति गलत धारणाहरू छन् ? | छन्  छैनन् | ०  १ |  |
|  | यदि छन् भने तिनिहरुको नाम लेख्नुहोस |  |  |  |

**खण्ड ४:खाना आवृत्ति प्रश्नावली**

**(सोध्नुहोस):** तपाईंले अन्तिम 24 घण्टा वा 7 दिनमा प्रत्येक खानाहरुको सूचीबाट कुन कुन खाना खानुभयो ?

| **सि.नं.** | **खानाहरुको समुह** | **के तपाईले पछिल्लो २४ घण्टामा निम्न खानेकुराहरु खानु भयो ?**  **०= खाएको छैन**  **१= खाएको छु** | **यदि हो भने¸ पछिल्लो २४ घण्टामा निम्न खाने कुराहरु कति पटक खानु भयो ? १¸२ वा ३ आदि ।** | **तपाईले पछिल्लो ७ देखि निम्न खाने कुराहरु खानु भयो ? ०= खाएको छैन१= खाएको छु** | **यदि हो भने¸ पछिल्लो ७ दिन देखि निम्न खानेकुराहरु कति पटक खानु भयो ?१¸२¸३¸४¸५¸६¸ वा ७ आदि ।** |
| --- | --- | --- | --- | --- | --- |
|  | रातो मासु¸ कलेजो¸माछा |  |  |  |  |
|  | भिटामिन सि युक्त खानाहरु-अमिलो फलफूलहरु |  |  |  |  |
|  | गेडागुडीहरु (बदाम¸राजमा¸ओखर¸  भटमास आदि) |  |  |  |  |
|  | दुधबाट उत्पादन भएका खाने  कुराहरु (मखन¸दही¸पनिर) |  |  |  |  |
|  | अन्न र जराहरुबाट आउने खानेकुराहरु (चामल¸ मकै¸ रोटी¸कोदो¸पिडालु¸आलु¸तरुल आदि) |  |  |  |  |
|  | अण्डा |  |  |  |  |
|  | भिटामिन ए युक्त फलफुलहरु आँप¸ गाजर¸ सखरखण्ड आदि |  |  |  |  |
|  | गाढा हरीयो सागपातहरु |  |  |  |  |

**खण्ड ५: एन्थ्रोपोमेट्रि बिवरण**

| नाप | 1^st^ | 2^nd^ |
| --- | --- | --- |
| उचाई (cm) |  |  |
| वजन (kg) |  |  |
| वि.एम.आई (BMI) |  |  |

**खण्ड ६: बायोकेमिकल परीक्षण**

| परीक्षण | 1^st^ gm/dl | 2^nd^ gm/dl |
| --- | --- | --- |
| हेमोग्लोबिन |  |  |

**References**

1. Of E, Education N, An W, On E, Of C, On IF, et al. University of Ghana http://ugspace.ug.edu.gh UNIVERSITY OF GHANA COLLEGE OF BASIC AND APPLIED SCIENCES CONSUMPTION OF IRON-RICH FOODS ON HAEMOGLOBIN LEVELS OF. 2015;(10442391).
